# Correlation of s-IgA and IL-6 Salivary with Caries Disease and Oral Hygiene Parameters in Children

**DOI:** 10.3390/dj8010003

**Published:** 2019-12-27

**Authors:** Giuseppe Lo Giudice, Fabiana Nicita, Angela Militi, Rossella Bertino, Marco Matarese, Monica Currò, Carmelo Salpietro Damiano, Carmen Mannucci, Gioacchino Calapai

**Affiliations:** 1 Department of Biomedical and Dental Sciences and Morphofunctional Imaging, University of Messina, 98100 Messina, Italy; fabin92@hotmail.it (F.N.); amiliti@unime.it (A.M.); lilro@hotmail.it (R.B.); matamarco94@gmail.com (M.M.); monica.curro@unime.it (M.C.); cmannucci@unime.it (C.M.); gioacchino.calapai@unime.it (G.C.); 2 Department of Adult and Childhood Human Pathology “Gaetano Barresi”, University of Messina, 98100 Messina, Italy; carmelo.salpietro@unime.it

**Keywords:** dental caries, s-IgA, IL-6

## Abstract

This study evaluates salivary immunoglobulin A (s-IgA) and interleukin 6 (IL-6) in saliva of children and its correlation to tooth decay severity. Fifty-nine patients were divided into two groups: caries free (A group) and caries active (B group). B group was investigated according to Mount and Monse indices. Mean salivary IgA rate between two groups (A 16.7 ± 4.5 mg/dL vs. B 21.8 ± 12.9 mg/dL) was not significant, while IL-6 rate (A 19.02 ± 5.3 pg/mL vs. B 30.2 ± 11.8 pg/mL) was statistically different. This study revealed that salivary IL-6 levels were significantly higher in children with active caries when compared with the caries-free group, while the s-IgA rate showed no significant differences between the two groups.

## 1. Introduction

The physiological composition of the oral ecosystem is the result of the competitive balance between oral microbiota and immune defensive system. An altered relationship between potential pathogenicity and the ability to self-repair and immune defense creates assumptions for the development of oral inflammatory diseases [1]. Saliva protective mechanism is necessary to reduce and prevent dental caries. Saliva contains a large number of antimicrobial proteins, for instance immunoglobulins such as immunoglobulin A (IgA), immunoglobulin M (IgM), and immunoglobulin G (IgG) and antimicrobial agents such as lactoferrin, lysozime, mucins, histatins, lactoperoxidases, and proline-rich glycoproteins. Most of these substances are present in low concentrations, but they act through a synergic effect in reducing bacterial growth and metabolism and being an effective network for oral defense [2,3,4].

A low level of salivary proteins is a risk factor for the development of dental caries [5,6]. Many studies, conducted to evaluate the role of salivary Ig (s-Ig) in different oral infections, demonstrated that IgA is the main specialized antibody isotype of the oral immune system [7]. 

The IgA secretory response is the first immune adaptive defense against *Streptococcus mutans*, considered the main causative bacteria of dental caries, acting through inhibition of bacterial adherence and reduction of streptococcus oral colonization [8,9,10,11]. However, the relationship between IgA levels and dental caries is still not clearly identified.

Caries can cause oral immune responses by inducing the production of different cytokines, interleukin 6 (IL-6), IL-1, IL-8 and tumor necrosis factor (TNF)-α. Moreover, high cytokine levels were found in different inflammatory states including periodontitis and oral lichen planus that can induce marginal inflammation [12]. The cytokine IL-6 is a pro- and anti-inflammatory molecule produced by T cells and macrophages that stimulate immune response [13]. Several studies showed a significant correlation between IL-6 salivary levels and dental caries, thus supporting the concept that caries disease is a chronic inflammatory condition [14,15,16,17]. 

The aim of this study was to evaluate the s-IgA and IL-6 levels in children with mixed dentition and their correlation with caries decay severity. The research hypothesis was to evaluate the possibility that an increase in IgA and IL-6 levels is statistically evident when comparing a group of patients affected by caries to a caries-free control group. 

A further hypothesis was to see if a correlation was present between IgAs/IL-6 levels and caries severity or presence of complication.

## 2. Materials and Methods

The study was carried out at the Department of Dentistry of Messina University and included 59 patients from 4 to 16 years old coming from the Department of Genetics and Paediatric Immunology. The procedures were conducted in accordance with the guidelines of the Helsinki Declaration. For inclusion in the study all subjects signed an informed consent. The study protocol was approved by the Ethics Committee of Azienda Ospedaliera Universitaria Messina, protocol 18/18 of 23 April 2018. The sample size was decided considering the probability of type-I error = 0.05 and a power of 0.95.

After all parents/tutors signed the informed consent their children were enrolled in the study; considering the non-invasive nature of the test carried out, a health record including anamnestic data was compiled by a single patient and intraoral and radiographic examinations (intraoral bitewing radiograph) were done [18]. Children with a history of systemic and autoimmune diseases or those who took antimicrobial agents during the last three months were excluded from the study.

The total number of Decayed, Missing, Filled Teeth (DMFT) index was collected for each single patient [19]. Based on the DMFT mean value the patients were divided into two groups:

A group—20 patients; caries-free control group (DMFT = 0);
B group—39 patients; caries active group (DMFT > 0).

To evaluate the severity of cavity lesions the B group was analyzed according to two other classifications:

1. Pulpal involvement, Ulceration, Fistula, Abscess (PUFA) index: used to quantify the severity and extension of the consequences of untreated caries in permanent and primary dentition [20]. Such a combination let us record the clinical consequences of untreated dental caries [21]. The PUFA index defines four different clinical stages of advanced caries and the score was calculated assigning one score for a single tooth with PUFA diagnostic criteria (Table 1). The score was identified by assigning a point per single tooth affected by complications.
2. Mount and Hume Site/Stage (Si/Sta) index: defines the severity of a carious lesion using topographic and dimensional criteria (Table 1) [22]. For statistical analysis this categorical index needed numerical correction. For each stage (1–4) a numerical value based on increased arithmetic progression was used. For each stage the numerical values were multiplied by the number of decayed teeth found and the weighted sum of all scores was calculated. Two subgroups, mildly active caries (MAC) and highly active caries (HAC), were identified using the range of scores respectively, 1–10 and >10.

### 2.1. Saliva Collection

Saliva collection was performed from 10:00 to 11:00 a.m. We recommended avoiding consumption of any food or drink and oral hygiene procedures for 90 min before saliva collection. For s-IgA assay the children were instructed to spit in sterile tubes once a minute for 10 min in order to sample whole saliva [12,23]. The IL-6 assay was performed by a trained operator on gingival crevicular fluid samples collected using sterile filter papers. Then the samples were immediately submitted for immunochemistry investigation. 

### 2.2. Evaluation of s-IgA in Saliva Samples by ELISA

In order to detect s-IgA, an enzyme-linked immunosorbent assay was performed in saliva samples collected from patients with and without caries, using the s-IgA ELISA kit from Cloud-Clone Corp in accordance with the manufacturer’s instructions. Saliva samples were centrifuged at 1000× *g* for 15 min at 4 °C and supernatants were used for the test after dilution (1/5000 in phosphate-buffered saline (PBS)). Briefly, 100 µL of standards or diluted samples were incubated in 96-well plates for 1 h at 37 °C. 

Then, before incubation, in each well a biotinylated detection antibody specific for s-IgA and avidin-horseradish peroxidase conjugate was added. Free components were washed away, and the substrate solution was added to each well. The addition of a sulfuric acid solution was used to end the enzyme-substrate reaction and the optical density (OD) was determined at a wavelength of 450 nm using a microplate reader (Tecan, Milan, Italy).

Both standards and samples were run in duplicate. The concentration of s-IgA in the samples was calculated by comparing the OD of the samples to the standard curve and reported in mg/dL.

### 2.3. Evaluation of IL-6 Salivary Levels in Saliva Samples by ELISA

IL-6 protein levels were measured in gingival crevicular fluid samples using the commercially available Human IL-6 Quantikine ELISA Kit (R&D Systems; Minneapolis, MN, USA) specific for human IL-6. Briefly, filter papers were unwrapped and introduced into a sterile test tube containing 0.5 mL phosphate-buffered saline (PBS). The tubes stood at 37 °C for 30 min. In order to facilitate extraction of the sample from the filter paper the samples were agitated every 5 min. All analyses were performed according to the manufacturer’s protocol. Absorbance was measured at 450 nm (correction wavelength set at 540 nm) by a Wallac 1420 Victor2 multi-label counter (Perkin-Elmer Life Sciences, Turku, Finland). 

### 2.4. Statistical Analysis

The numerical data (DMFT, PUFA, IgA, and IL-6) were expressed as mean value ± standard deviation (SD) and the categorical variables (caries severity) as numbers and percentages. 

Variables examined did not present normal distribution as verified by the Kolmogorov–Smirnov test. Consequently, the non-parametric approach was used.

Statistical comparisons between caries-free and caries active groups were carried out using the Chi-squared test for categorical variables and the Mann–Whitney test for numerical parameters. The Mann–Whitney test was applied only for caries active group in order to compare two subgroups, MAC and HAC, regarding IgA and IL-6 concentrations. A *p*-value < 0.05 was considered statistically significant. Statistical analysis was performed using SPSS version 17.0 computer software. 

## 3. Results

The mean s-IgA and IL-6 concentrations in saliva in A group were respectively 16.7 ± 4.5 mg/dL and 19.02 ± 5.3 pg/mL, whereas for the B group such parameters were 21.8 ± 12.9 mg/dL for IgA and 30.2 ± 11.8 pg/mL for IL-6 (Table 2). The Mann–Whitney test did not show statistically significant differences regarding the IgA concentrations between the two groups examined, while the difference was statistically highly significant for IL-6 levels (*p*-value < 0.001). 

Thirty-nine patients in the B group presented a mean DMFT value of 0.25 ± 0.24 with PUFA = 0.02 ± 0.05 in the primary dentition and the mean DMFT of 0.06 ± 0.08 with PUFA = 0 in the permanent dentition (Table 2).

In caries active group seven of 39 patients with PUFA > 0 presented mean s-IgA and IL-6 concentrations of 32.2 ± 20.8 mg/dL and 47.1 ± 15.9 pg/mL, respectively, while 32 children with PUFA = 0 demonstrated salivary IgA levels of 19.5 ± 9.4 mg/dL and IL-6 levels of 26.5 ± 6.6 pg/mL. The Mann–Whitney test showed statistically significant difference only for IL-6 concentrations between two groups in the exam (*p*-value < 0.001) (Table 3).

IgA and IL-6 mean concentrations in the B group were divided into two subgroups MAC and HAC (Table 3). The Mann–Whitney test did not reveal any significant difference regarding the IgA and IL-6 concentrations between the two subgroups. 

## 4. Discussion

The hypothesis of the infectious nature of caries disease supposes that immune mechanisms, in particular, salivary IgA, intervene in oral defense processes. 

Nevertheless, the correlation between s-IgA and dental caries is still not clearly defined. Many studies showed an increase in the level of s-IgA during caries process compared to the caries-free subjects [12,24,25,26]. 

Other findings showed that higher levels of salivary IgA were related to reduction of caries activity [27,28]. These results seem to demonstrate that the s-IgA production provides a local defense in connection with dental infection.

Other researches demonstrated a significant reverse correlation between s-IgA and carious activity [29]. 

This variability leads us to conclude that the quantity of s-IgA and the presence of carious lesions are not correlated [30]. According to Omar et al. [31], IgA levels were higher than those of caries-free subjects. Nevertheless, the same study revealed that children with a low experience of caries showed higher s-IgA levels than those of the caries-free control group. Thus, IgA could increase as a protective response of a low caries experience. This hypothesis has already been suggested by Brandtzaeg [32], who reported that a large number of cariogenic bacteria in untreated carious lesions could stimulate the local immune response with a secondary increase of salivary IgA.

Our study confirms a high variability of data and did not show a significant difference between the s-IgA mean concentration of caries-free and caries active groups; although higher s-IgA levels were found in caries active patients compared to caries-free patients (21.8 ± 12.9 mg/dL vs. 16.7 ±4.5 mg/dL). These findings are in agreement with many researches that highlight the absence of any correlation between dental caries and increased IgA levels [33,34,35].

Moreover, our results showed that the IL-6 salivary levels were highly significant in caries active group (30.2 ± 11.8 pg/mL). This result is in agreement with Gornowicz et al. [14] as well as Menon et al. [17], who showed that IL-6 concentration was higher in caries active children compared to the control group. 

In our research these outcomes are confirmed by significant evidence between subjects with PUFA > 0 and those with PUFA = 0; in fact, the IL-6 concentration was absolutely higher in children who developed odontogenic infection (47.1 ± 15.9 pg/mL vs. 26.5 ± 6.6 pg/mL). Therefore, the IL-6 production appears to be associated with active caries. Our study did not confirm any correlation between dental caries extension and s-IgA and IL-6 levels. In fact, the mean s-IgA and IL-6 concentrations are not significantly different between MAC and HAC of B group.

Considering that levels of salivary inflammatory cytokines seem to be divided in different body fluids, IL-6 could be considered as valid predictor for the presence of dental inflammation [36,37].

In addition, in the detection of children with inflammatory diseases, the salivary interleukin tests can be used only in children with healthy dentition [38,39].

## 5. Conclusions

Tooth decay is due to the imbalance between demineralization induced by biofilm and oral defensive capabilities which include immune and inflammatory responses. The salivary IgA activation mode and protective mechanisms against caries disease are still not fully explained.

Our study does not show any significant correlation between salivary immunoglobulin A levels and dental caries. On the contrary, IL-6 is correlated to dental caries, especially when caries are in an active state and associated to infectious consequences. In conclusion, further insights are necessary in order to better evaluate cytokine role in pulp inflammatory processes.

## Figures and Tables

**Table 1:** Dental caries classifications.

PUFA Index	Mount and Hume Si/Sta
**P**: The involvement of the pulp tissue is registered whenever the pulp chamber is exposed, or the coronal tooth structure has been compromised by carious process. **U**: Ulceration of soft tissues (tongue, buccal mucosa) caused by sharp edges of the crown of misplaced teeth or by root fragments. **F**: Fistula is registered when there is a pus releasing tubular tract originating from a tooth with pulpal involvement. **A**: Abscess is registered when there is a pus containing swollen area originating from a tooth with pulpal involvement.	**Position** **Site 1**: occlusal grooves of the posterior teeth and smooth surfaces of the anterior teeth; **Site 2**: interproximal surfaces, contact points; **Site 3**: cervical third and exposed roots. **Size** **Stage 1**: small size, minimum dentin involvement; **Stage 2**: medium size, moderate dentin involvement; **Stage 3**: large dimensions with significant dentin involvement; **Stage 4**: very extensive lesions with significant loss of tooth structure.

**Table 2:** A and B groups. Mean values of DMFT, PUFA, salivary immunoglobulin A (s-IgA) (mg/dL), and interleukin 6 (IL-6) (pg/mL).

GROUP	DMFT	DMFT	PUFA	PUFA	IgA Mean	IL-6 Mean
A	0	0	0	0	16.7 ± 4.5	19.02 ± 5.3
B	0.06 ± 0.08	0.25 ± 0.24	0	0.02 ± 0.05	21.8 ± 12.9	30.2 ± 11.8

**Table 3:** B group (39 children). Mean values of DMFT, PUFA, s-IgA (mg/dL), and IL-6 (pg/mL).

INDEX	N.	IgA Mean mg/dL	*p*-Value	IL-6 Mean pg/mL	*p*-Value
PUFA = 0	32	19.5 ± 9.4	0.175	26.5 ± 6.6	0.001
PUFA > 0	07	32.2 ± 20.8		47.1 ± 15.9	
Mildly Caries Active	24	20.8 ± 9.5	0.977	27.2 ± 7.1	0.601
Highly Caries Active	15	23.3 ± 17.2		34.9 ± 16.06	
